# Adolescent well‐being amid the COVID‐19 pandemic: Are girls struggling more than boys?

**DOI:** 10.1002/jcv2.12027

**Published:** 2021-08-03

**Authors:** Thorhildur Halldorsdottir, Ingibjorg Eva Thorisdottir, Caine C. A. Meyers, Bryndis Bjork Asgeirsdottir, Alfgeir Logi Kristjansson, Heiddis B. Valdimarsdottir, John P. Allegrante, Inga Dora Sigfusdottir

**Affiliations:** ^1^ Department of Psychology Reykjavik University Reykjavik Iceland; ^2^ Icelandic Center for Social Research and Analysis Reykjavik Iceland; ^3^ Department of Social and Behavioral Sciences School of Public Health West Virginia University Morgantown West Virginia USA; ^4^ Population Health Science and Policy Icahn School of Medicine at Mount Sinai New York City New York USA; ^5^ Department of Health and Behavior Studies, Teachers College Columbia University New York City New York USA; ^6^ Department of Sociomedical Sciences Mailman School of Public Health Columbia University New York City New York USA

**Keywords:** adolescence, COVID‐19, depressive symptoms, gender, mental health, pandemic, social media

## Abstract

**Background:**

Differential effects of the coronavirus SARS‐CoV‐2 (COVID‐19) pandemic and associated public restrictions on adolescent girls and boys are emerging but have not been elucidated. This study examined gender differences across broad indicators of adolescent well‐being during the COVID‐19 pandemic in Iceland, and explored potential explanations for these differences.

**Methods:**

In total, 523 youth (56.5% girls) born in Iceland in 2004 completed measures on mental health problems (depressive symptoms, anger and suicide attempts) and measures designed for this study to assess broad indicators of adolescent well‐being (e.g., day‐to‐day life, academic performance, family and peer relationships, and mental and physical health) and behavioral changes during the COVID‐19 pandemic. Mental health problems during the pandemic were compared to expected scores based on nationwide ratings of same‐aged peers in 2018.

**Results:**

Although both boys and girls appeared affected, girls reported a greater negative impact across all the broad indicators of well‐being and behavioral change during COVID‐19 than boys, and their depressive symptoms were above and beyond the expected nationwide scores (*t*(1514) = 4.80, *p < *.001, Cohen's *d* = 0.315). Higher depressive symptoms were associated with increased passive social media use and decreased connecting with family members via telephone or social media among girls, and decreased sleeping and increased online gaming alone among boys. Concern about others contracting COVID‐19, changes in daily and school routines, and not seeing friends in person were among the primary contributors to poor mental health identified by youth, particularly girls.

**Conclusions:**

Adolescents were broadly negatively affected by the COVID‐19 pandemic and accompanying restrictions; however, this negative impact was more pronounced in girls. The findings suggest that a steady routine and remaining socially connected may help youth cope with the uncertainty and social restrictions associated with a pandemic. Moreover, healthcare providers, teachers, and other professionals should pay close attention to depressive symptoms among girls during a pandemic.

## INTRODUCTION

Adolescence is characterized by an increased desire for independence, autonomy, and reliance on peer connections for emotional support and social development, coupled with heightened sensitivity to stress exposure associated with pubertal development (Bailen et al., 2019; Ellis & Zarbatany, 2017; Orben et al., 2020). As such, the combination of concerns of the contagion (COVID‐19) SARS‐CoV‐2 and measures to contain its transmission (e.g., forced physical distance from friends, and confinement to home) are likely to negatively affect the well‐being of adolescents. Notably, the COVID‐19 pandemic intersects with rising prevalence of mental health problems and harmful behaviors among adolescents (Collishaw, 2015; Thorisdottir et al., 2017). The combination of these factors has been described as “the perfect storm,” resulting in rising concerns by healthcare providers and researchers worldwide about a mental health pandemic among adolescents and young adults in the aftermath of the COVID‐19 pandemic (Golberstein et al., 2020; Orben et al., 2020).

**Key points**

- Emerging evidence suggests that adolescent mental health has been negatively impacted during the COVID‐19 pandemic; however, the impact on the broader indicators of well‐being remain unknown.
- The study examined gender differences in several indicators of adolescent well‐being during COVID‐19 and potential explanations for observed differences.
- To mitigate the effect of COVID‐19, the findings suggest that policy makers and parents should attempt to maintain school routines as similar as possible to the pre‐COVID‐19 period, and encourage youth—both boys and girls—to remain socially connected through telephone and online interactions, and physically active, despite being isolated at home.
- Healthcare providers, teachers and other professionals need to especially monitor depressive symptoms and the well‐being of girls during COVID‐19 and beyond.

### Gender differences in adolescents' response to the COVID‐19 pandemic

Emerging evidence indicates that adolescent girls are disproportionally negatively affected by the pandemic compared to adolescent boys (Duan et al., 2020; Ellis et al., 2020; Hafstad et al., 2020; Kang et al., 2020; Kapetanovic et al., 2021; Magson et al., 2020; Ravens‐Sieberer et al., 2021; Thorisdottir et al., 2021; Wright et al., 2021; Zhou et al., 2020). Girls have been found to present with higher levels of depressive and anxiety symptoms (Chen et al., 2020; Ellis et al., 2020, 2020; Hafstad et al., 2020; Kapetanovic et al., 2021; Magson et al., 2020; Thorisdottir et al., 2021; Wright et al., 2021; Zhou et al., 2020) and increased tension, anger, and confusion (Kang et al., 2020) compared to boys during the pandemic. Researchers have also postulated that the pandemic may lead to an increase in suicides or suicidal ideation among adolescents (Fegert et al., 2020; Hoekstra, 2020). The limited available studies examining the prevalence rates of confirmed suicides and admission to psychiatric clinics suggest that this is not the case (Isumi et al., 2020; Mourouvaye et al., 2020). However, many suicide attempts never come to the attention of healthcare providers and are therefore not documented in registries or hospital records (Kidger et al., 2012). Accordingly, research leveraging self‐reported suicidal behavior is needed to better understand whether a true increase has occurred during the pandemic.

Few studies to date have examined gender differences in other indicators of well‐being among adolescents during the pandemic. Consistent with the reported mental health findings, recent studies indicate girls reported less overall life satisfaction and increased conflict with parents during the COVID‐19 pandemic compared to boys (Kapetanovic et al., 2021; Magson et al., 2020). However, further elucidation on the broad‐scale effect of COVID‐19 on adolescent girls and boys is needed. For example, it is unclear whether there are gender differences in academic performance, familial and peer relationships, and physical health. Determining the broad‐scale psychosocial consequences of the COVID‐19 pandemic has important clinical implications. Initial impressions from adolescents of the pandemic's effect may shed light on both the potential effect of the pandemic and what intervention efforts might prove most effective in prevention or amelioration. These can then be followed up with prospective longitudinal studies with comparative data collected prior to the pandemic that can be used to map the potential effect of COVID‐19 on adolescents.

### What could be contributing to gender differences?

In terms of explaining gender differences in mental health problems, it has been posited that pandemic control restrictions (e.g., online schooling, social distancing) impede adolescents girls' ability to rely on their social network for emotional support in stressful times (Magson et al., 2020). Furthermore, research has suggested that girls engage in higher levels of sedentary behavior and increased social media use/screen time and increased time sleeping compared to boys (Dunton et al., 2020; Ellis et al., 2020; Kang et al., 2020; Moore et al., 2020). All of these behaviors have been linked to an increase in mental health problems. In contrast, some studies find that boys also report higher levels of vigorous physical activity compared to girls, which may confer protection against negative mental health outcomes (Chen et al., 2020; Dunton et al., 2020; Kang et al., 2020; Schmidt et al., 2020; Wright et al., 2021).

As detailed above, the COVID‐19 pandemic has given rise to circumstances in which negative consequences are more likely to occur. However, it is important to note that there is also potential for positive consequences to occur after such large‐scale societal stressors (Bruining et al., 2020). For instance, an increase in happiness was observed in adolescents ages 14–15 years old following the Icelandic economic crisis in 2008 (Gudmundsdóttir et al., 2016). In terms of the COVID‐19 pandemic, some families may develop strong connections and cohesion by spending more time together (Fegert et al., 2020). Furthermore, staying at home may relieve stress from peer and academic difficulties experienced at school (Fegert et al., 2020; Hoekstra, 2020). This in turn may mitigate the potentially negative impact of the COVID‐19 crisis. As previous research indicates, it is highly likely that there are gender differences in what aspects of the pandemic are considered by youth as negatively or positively affecting their well‐being. In order to develop effective prevention initiatives, it is vital to determine which factors contribute to negative and positive mental health for whom in the face of adversity.

### The present study

The current study is the first to examine perceived gender differences in broad‐scale of adolescent well‐being (i.e., academic performance, day‐to‐day life, family and peer relationships, and mental and physical health) during the COVID‐19 pandemic and explore potential explanations for these differences. First, we examined gender differences in the perceived effect of COVID‐19 and associated public health restrictions on broad‐scale measures of adolescent functioning. Second, we compared the prevalence rates of depressive symptoms, suicide attempts, and anger among adolescent girls and boys during COVID‐19 to same‐age peers prior to the pandemic. Third, we investigated changes in daily behaviors during the pandemic, compared these changes between girls and boys, and then examined their association with mental health outcomes. Finally, we explored gender differences in perceived contributions to poor or good well‐being during the pandemic in adolescents.

## MATERIALS AND METHODS

### Participants

Participants comprised a subsample of 523 youth (54.9% girls) from the LIFECOURSE (*L*ongitudinal *I*nvestigation *F*or *E*pidemiologic *C*auses and *OU*tcomes *R*i*S*ing in *E*arly Childhood and Adolescence) study (*n* = 2378) (Halldorsdottir et al., 2020). The LIFECOURSE study has been funded by Project Grants (206580 and 217612) by the Icelandic Research Fund and a Research Consolidator Grant from the European Research Council (ERC‐CoG‐2014‐647860). Eligible participants for the LIFECOURSE study were all children born in Iceland in 2004 and resided in Iceland in 2017. Recruitment for the current study involved sending an invitation letter home and calling families from the original LIFECOURSE study, and advertising the study in secondary schools. Of the families participating in the original LIFECOURSE study, 1930 were residing in Iceland and had a valid postal address at the time of data collection for this study. The study was approved by the National Bioethics Committee of Iceland (11‐078) and registered with the Personal Protection Authority.

### Measures

#### Background variables

Participants reported their gender (1 = *boy*, 2 = *girl*, 3 = *other*). To gauge socioeconomic status, participants provided information on parental employment status (1 = *full‐time employment*, 2 = *part‐time employment*, 3 = *other*) and household status (1 = *living with two parents/caregivers*, 2 = *other*). Youth also reported the use of psychotropic medication for depression, anxiety, inattention, and hyperactivity. The use of psychotropic medication was dichotomized (0 = *no psychotropic medication*, 1 = *using psychotropic medication*).

### Mental health outcomes

Since 1998, the Icelandic Centre for Social Research and Analysis (ICSRA) has conducted biennial, population‐based surveys among 10‐20‐year‐old youth in Iceland that provide information from over 80% of the population (Sigfusdottir et al., 2020). Given the overlap in the mental health measures used in the ICSRA studies and this study, the prevalence rates from same‐aged peers from the ICSRA administration in 2018 (1554 girls and 1445 boys) served as expected scores of the prevalence of mental health problems.

#### Depressive symptoms and anger

The depressed mood and anger subscales of the Symptom Checklist‐Revised (SCL‐90) (Derogatis et al., 1975) were used to measure depressive symptoms and anger in the previous week. Participants rated how often 10 items pertaining to depressed mood and 5 items on anger applied to them in the past week on a four‐point Likert scale (0 = *almost never* to 3 = *often*). A composite score of these items was created, with higher scores suggesting higher levels of depressive symptoms (range, 0–30) and anger (range, 0–15). The scales demonstrated excellent psychometric properties (Derogatis & Unger, 2010) (depressive symptoms: *α* = .91; anger: *α* = .83). The SCL‐90 is a well‐established and widely used measure in clinical settings, as well as population‐based research. It has historically been used to examine psychopathological dimensions; however, research has shown that the SCL‐90 performs well in detecting psychopathology among adolescents (Rytilä‐Manninen et al., 2016; Wiznitzer et al., 1992). Previous work from our group has also shown that the subscales of the depression dimension of the SCL‐90 administered at a nationwide level corresponds to the prevalence of visits to pediatric psychiatrists and clinical child psychologists (Sigfusdottir et al., 2008).

#### Suicide attempts

Participants were asked to respond “yes” or “no” to the question: “Have you attempted suicide in the past year?” The timeframe of the items was confined to the past year in order to capture changes in its prevalence during the COVID‐19 from previous years. This method was chosen as previous research indicates that many suicide attempts never come to the attention of healthcare workers (Kidger et al., 2012). Further, there is evidence to suggest that adolescents are more likely to report suicide attempts under conditions of anonymity (Safer, 1997). Therefore, self‐reported indicators of suicide attempts may be more reliable than registry reports to measure its prevalence among this population.

### Pandemic‐related survey items

Survey items were developed for this study to evaluate adolescents' responses to the COVID‐19 pandemic. The survey was developed after the onset of the COVID‐19 pandemic in Iceland and developed based on existing international surveys on the effect of COVID‐19 on youth at the time (Pearcey et al., 2020). Each measure is described here below and the full text of these survey items is provided in the online supporting information material (Tables S1 and S2).

#### Broad‐scale effect of COVID‐19

Respondents rated the potential effects COVID‐19 had on their overall day‐to‐day life, academic performance, relationship with family and peers, and physical and mental health using a five‐point Likert scale (1 = *much worse* to 5 = *much better*). To obtain a more nuanced view of the perceived effect of COVID‐19 on adolescents, we examined these responses in two ways: as a continuous and binary variable. For the latter, response for each was dichotomized based on whether it was considered to be negatively (0 = *better or no change*, 1 = *worse or much worse*) and positively affected (0 = *worse or no change*, 1 = *better or much better*) by COVID‐19.

#### Behavioral change during COVID‐19

Survey items captured COVID‐19 specific changes in the adolescents' daily habits. Respondents were asked to rate on a five‐point Likert scale (1 = *much less time* to 5 = *much more time*) how much time they spend on a list of 16 activities now compared to prior to the pandemic, such as sleeping, engaging in physical activity, using social media, and meeting up with friends or family.

#### Negative and positive effect on mental health due to COVID‐19

Survey items assessed what aspects of the COVID‐19 pandemic were contributing to poor and good mental health outcomes. Participants were provided with a list of nine potentially negative factors (e.g., not seeing friends in person and worrying about oneself and others getting COVID‐19) and 11 potentially positive factors associated with the pandemic (e.g., more time to sleep or less stress due to school) and asked to mark all that they considered having an effect on their mental health. In the current study, we compared how many negative and positive factors girls and boys endorsed, as well as examined gender differences in what factors were considered to contribute negatively or positively affect adolescent mental health.

### Procedure

All participants from the LIFECOURSE study were invited to participate in this study. For each participant, one caregiver provided informed consent via electronic/bank ID and the adolescent provided assent prior to entering the survey in RedCap. The survey inquiring about well‐being during COVID‐19 was administered from October 14, 2020 to April 16, 2021. Participants in the current study completed self‐report survey battery through the RedCap platform. During this 7‐month period, Iceland underwent two waves of COVID‐19. The 14‐day incidence rate of infections during this time was on average 49.5 per 100,000 inhabitants. To limit transmission of SARS‐CoV‐2, mandatory social restrictions of 20 individuals or less gathering at once was in place for 6 months during this time. These restrictions resulted in secondary schools switching to online instruction, organized group and sport activities were suspended, and restaurants and bars were temporarily closed for a large portion of this time.

### Statistical analyses

Proportions and *χ* ^2^ tests were used to examine gender differences in demographic characteristics.

To address our research aims, generalized linear regression models were used to document gender differences for each broad‐scale measure of well‐being (i.e., academic performance, day‐to‐day life, family and peer relationships, and mental and physical health). Logistic regression models were used to determine gender differences in the number of areas considered to be negatively or positively affected by COVID‐19. Second, gender differences in mental health problems during COVID‐19 were examined. Independent *t* tests and *χ* ^2^ tests were used to compare mental health problems during the pandemic to expected scores based on nationwide averages (depressive symptoms and anger) or percentages (suicide attempts) of mental health problems obtained on same‐aged peers in 2018. Third, generalized linear regression analyses was used to examine gender differences in each indicator of behavioral change. Then, for the mental health problems we found to be exacerbated by the pandemic, we examined whether the behavioral change indicators (all entered simultaneously) were associated with the mental health outcome using generalized linear regression analyses. Finally, logistic regression models were used to determine gender differences in the factors the respondents identified as negatively or positively affecting their well‐being during COVID‐19. Given its association with mental health problems. All analyses were adjusted for parental employment status (1 = full‐time employment, 2 = part‐time employment, 3 = other) and household status (1 = living with two parents/caregivers, 2 = other) was included as a covariate in all analyses.

Cohen's *d* was calculated using the *esc* R package (Lüdecke, 2018) to gauge the strength of the findings. The unstandardized regression coefficient and standard deviation for continuous outcomes and OR and standard error for binary outcomes were used to calculate the Cohen's *d*. A positive value indicated an increase in the means or prevalence rates among girls compared to boys, while a negative value indicated a decrease. For hypothesis testing, the *α* value for significance testing was set to .05. All analyses and visualization of the findings were conducted in R.

## RESULTS

Characteristics of the total sample and by gender are provided in Table 1. Of the 523 participants, 54.9% identified as female (*n* = 287), 41.5% as male (*n* = 217), 0.5% as non‐binary (*n* = 2), and 0.6% did not complete this survey item (*n* = 17). Youth identifying as non‐binary were excluded from the gender analyses, given the small sample size of the group. No gender differences were noted in parental employment, household status, or use of psychotropic medication (*p* > 0.199).

**TABLE 1:** Demographic and clinical characteristics of the sample

Characteristic	Total, *n* (%)	Girls, *n* (%)	Boys, *n* (%)	*p*
Sample size	523	287 (54.9)	217 (41.5)	
Maternal employment				.199
Full‐time employment	366 (70.0)	164 (75.6)	199 (69.3)	
Part‐time employment	77 (14.7)	21 (9.7)	42 (14.6)	
Other	80 (15.3)	32 (14.7)	46 (16.0)	
Paternal employment				.900
Full‐time employment	434 (83.0)	186 (85.7)	244 (85.0)	
Part‐time employment	67 (12.8)	21 (9.7)	31 (10.8)	
Other	22 (4.2)	10 (4.6)	12 (4.2)	
Two‐parent household	399 (76.3)	168 (77.4)	227 (79.1)	.732
Psychotropic medication	80 (17.4)	29 (15.2)	51 (19.2)	.317

*Note:* Participants who identified as non‐binary gender (*n* = 2) or chose not to answer the gender question (*n* = 17) were not included in the girls or boys categories. In terms of psychomedication, there were participants who did not complete the survey item during the administration (*n* = 62).

When comparing youth participating in the current study to the general Icelandic population of 16‐ to 17‐year‐olds, significant differences in terms of gender emerged, with a higher percentage of girls participating in this study (54.9%) than in the general population (49.3%) (*χ* ^2^(1) = 11.264, *p* = .001). However, the percentage of participants living within a two‐caregiver household (76.3%) and enrolled in secondary school (95.4%) in the current sample were comparable to the general Icelandic population (74.6%; *χ* ^2^(1) = 0.755, *p* = 0.385 and 93.6%; *χ* ^2^(1) = 1.195, *p* = 0.274) (prevalence rates for the general population were derived from Thorisdottir et al., 2021 and the Icelandic Statistics Bureau).

### Broad‐scale effect of the pandemic

Regression models were used to determine differences in the perceived effect of COVID‐19 on broad‐scale indicators of well‐being, specifically academic performance, day‐to‐day life, family relationships, peer relationships, mental health, and physical health. A large proportion of youth rated their functioning across these areas as unchanged during the pandemic (see Figure S1 in the supporting information material for the distribution of responses by gender). In terms of gender differences, however, girls were significantly more likely than boys to consider the pandemic to have negatively influenced their day‐to‐day life (*b* = −0.503, SE = 0.090, *p < *.001, Cohen's *d* = −0.246), physical (*b* = −0.192, SE = 0.088, *p = *.029, Cohen's *d* = −0.096) and mental health (*b* = −0.470, SE = 0.092, *p < *.001, Cohen's *d* = −0.224). No significant gender differences were noted in academic performance, relationship with family or friends or the family's financial status (Table 2).

**TABLE 2:** Gender differences in broad‐scale indicators of well‐being during the COVID‐19 pandemic and a comparison of the percentage of youth who rated these broad‐scale indicators of well‐being as being negatively or positively affected by the pandemic

					Negative				Positive			
	*B*	SE	*p*	Cohen's *d*	OR	Lower CI	Higher CI	Cohen's *d*	OR	Lower CI	Higher CI	Cohen's *d*
Day‐to‐day life	−0.503	0.090	.000	−0.246	2.843	1.967	4.135	0.576	0.498	0.266	0.916	−0.385
Academic performance	−0.129	0.095	.178	−0.059	1.651	1.141	2.402	0.276	1.400	0.832	2.400	0.185
Family relationship	0.087	0.081	.286	0.047	1.425	0.836	2.479	0.195	1.769	1.138	2.788	0.314
Peer relationship	−0.051	0.091	.575	−0.025	1.480	1.009	2.183	0.216	1.168	0.710	1.945	0.086
Physical health	−0.192	0.088	.029	−0.096	1.798	1.249	2.601	0.324	0.991	0.545	1.826	−0.005
Mental health	−0.470	0.092	.000	−0.224	2.998	2.062	4.395	0.605	0.943	0.488	1.847	−0.032

*Note:* The analyses were adjusted for parental employment status and household status.

**FIGURE 1:**
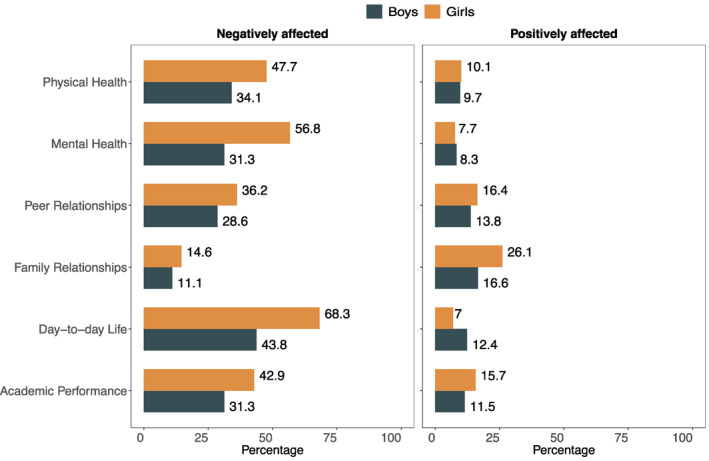
Percentage of adolescent girls and boys who rated physical and mental health, family and peer relationships, day‐to‐day life, and rated academic performance being negatively or positively affected by COVID‐19 compared to the period prior to the pandemic

Gender differences also emerged in the comparison of the percentage of youth who rated these broad‐scale indicators of well‐being as being negatively or positively affected by the pandemic (Figure 1). Specifically, compared to boys, girls were significantly more likely to report that the COVID‐19 pandemic had a negative impact on their day‐to‐day life (OR = 2.843, 95% CI 1.967–4.135, *p* < .001, Cohen's *d* = 0.576), academic performance (OR = 1.651, 95% CI 1.141–2.402, *p = *.008, Cohen's *d* = 0.276), peer relationships (OR = 1.480, 95% CI 1.009–2.183, *p = *.046, Cohen's *d* = 0.216), and mental (OR = 2.998, 95% CI 2.062–4.395, *p < *.001, Cohen's *d* = 0.605) and physical health (OR = 1.762, 95% CI 1.249–2.601, *p* = .002, Cohen's *d* = 0.324) and that it had a positive impact on their family relationship (OR = 1.769, 95% CI 1.138–2.788, *p = *.012, Cohen's *d* = 0.314). On the other hand, boys were significantly more likely than girls to rate their overall day‐to‐day life (OR = 0.498, 95% CI 0.266–0.916, *p* = .026, Cohen's *d* = −0.385) as positively affected by the pandemic. No significant gender differences were noted in terms of the perceived positive or negative effect of COVID‐19 on the other indices of broad‐scale functioning (*p* > .05).

### Mental health problems

**FIGURE 2:**
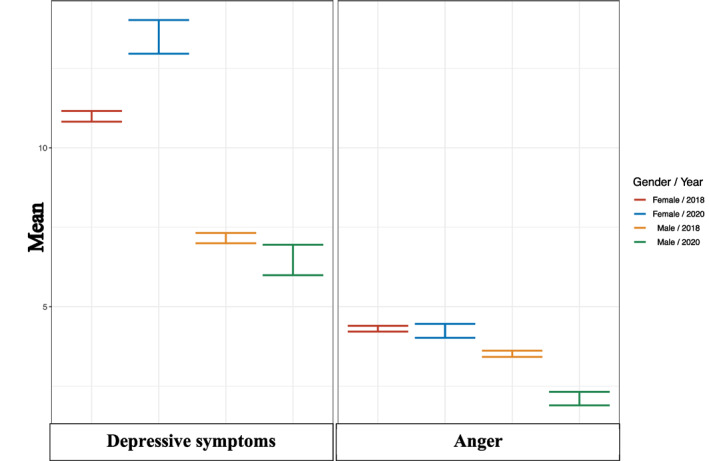
Mean depressive and anger symptoms among 16‐year‐old girls (depicted in blue) and boys (depicted in green) during COVID‐19 compared to same age peers prior to the pandemic (girls depicted in red and boys in yellow). Error bars reflect standard errors and the expected scores were derived from on nationwide administered school surveys in Iceland in 2018

Consistent with the poor mental health ratings among girls noted above, there was a significant increase in depressive symptoms among girls during the pandemic (*M* = 13.49, *SD* = 8.91) compared to the expected scores (*M* = 10.99, *SD* = 7.70; *t*(1514) = 4.80, *p < *.001, Cohen's *d* = 0.315). Interestingly, no differences in depressive symptoms were noted between 16‐year‐old boys during COVID‐19 (*M* = 6.46, *SD* = 7.03) compared to the expected score (*M* = 7.16, *SD* = 6.91, *t*(1272) = 0.905, *p = *0.450, Cohen's *d* = −0.101; Figure 2).

No differences were observed in the prevalence rates of girls (5.3%) or boys (4.7%) who had attempted suicide within the last 12 months during the pandemic compared to the prevalence rates among 16‐year‐old girls (4.5%) and boys (4.4%) in 2018 (*χ* ^2^(1) = 0.323, *p = *0.570 and *χ* ^2^(1) = 0.018, *p = *0.894). Similarly, no differences in measures of anger were noted among either adolescent girls (*M* = 4.24, *SD* = 3.16) or boys (*M* = 2.89, *SD* = 3.67) during the pandemic compared to the expected ratings (girls: *M* = 4.31, *SD* = 3.66, boys: *M* = 3.52, *SD* = 3.52; Figure 2).

### Behavioral changes due to COVID‐19

Compared to boys, girls reported spending more time sleeping (*b* = 0.219, SE = 0.099, *p = *.027, Cohen's *d* = 0.099), watching TV (*b* = 0.210, SE = 0.088, *p = *.018, Cohen's *d* = 0.106), talking with friends on the phone (*b* = 0.279, SE = 0.092, *p = *.003, Cohen's *d* = 0.135) and via social media (*b* = 0.407, SE = 0.089, *p < *.001, Cohen's *d* = 0.205), using social media to look at the profiles of friends (*b* = 0.252, SE = 0.085, *p = *.003, Cohen's *d* = 0.133) and people they do not know (*b* = 0.384, SE = 0.087, *p < *.001, Cohen's *d* = 0.198), using the Internet for something else than social media or gaming (*b* = 0.191, SE = 0.084, *p = *.023, Cohen's *d* = 0.101) and using the computer for something else than browsing online and playing computer games (*b* = 0.235, SE = 0.105, *p = *.026, Cohen's *d* = 0.100), and watching the news (*b* = 0.373, SE = 0.087, *p < *.001, Cohen's *d* = 0.192) (see Table S3 in the supporting information material). They also reported spending less time with friends (*b* = −0.231, SE = 0.102, *p = *.024, Cohen's *d* = −0.101) than boys.

Boys, on the other hand, endorsed spending more time playing computer games online alone (*b* = −0.291, SE = 0.093, *p = *.002, Cohen's *d* = −0.140) and with others (*b* = −0.338, SE = 0.096, *p < *.001, Cohen's *d* = −0.158), as well as playing offline games (*b* = −0.225, SE = 0.083, *p = *.007, Cohen's *d* = −0.121). See Figure S2 in the supporting information for the distribution of answers by gender.

Given the increase in depressive symptoms among girls, we next examined what self‐reported behavioral changes during the COVID‐19 pandemic were associated with depressive symptoms. When including both girls and boys in the analyses, increased passive social media use in the form of looking at strangers' social media profiles (*b* = 1.767, SE = 0.616, *p = *.004, Cohen's *d* = 0.128), and decreased online gaming with others (*b* = −1.573, SE = 0.671, *p = *.020, Cohen's *d* = −0.104) and connecting with family members over the phone or social media (*b* = −1.795, SE = 0.586, *p = *.002, Cohen's *d* = −0.137) were associated with increased depressive symptoms.

Stratifying by gender, increased time engaging in passive social media usage in the form of looking at a friend's social media profile (*b* = 1.936, SE = 0.958, *p = *.044, Cohen's *d* = 0.090) and decreased time connecting with family members over the phone or social media (*b* = −2.143, SE = 0.778, *p = *.006, Cohen's *d* = −0.123) were associated with increased depressive symptoms among adolescent girls. For adolescent boys, decreased sleep (*b* = −1.396, SE = 0.642, *p = *.031, Cohen's *d* = −0.097), online gaming with others (*b* = −1.839, SE = 0.849, *p = *.032, Cohen's *d* = −0.096) and increased online gaming alone (*b* = 2.311, SE = 0.863, *p = *.008, Cohen's *d* = 0.119), and engaging with friends via social media (*b* = 2.323, SE = 1.105, *p = *.037, Cohen's *d* = 0.094) were associated with higher depressive symptoms.

### Negative and positive effects of COVID‐19 on adolescent mental health

**FIGURE 3:**
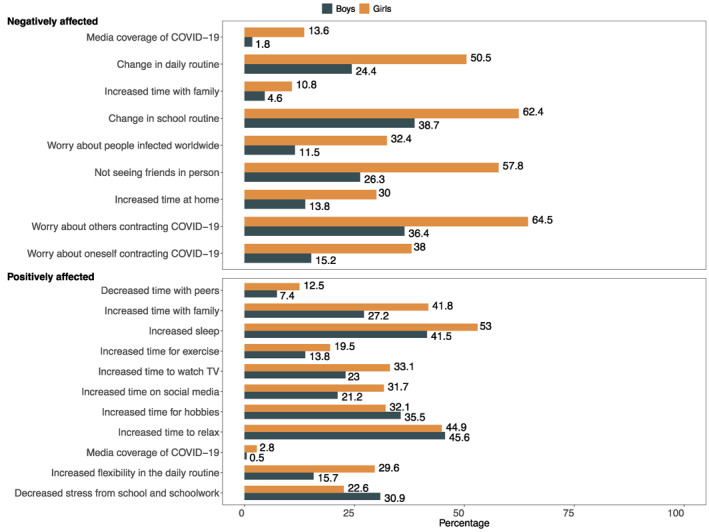
Gender differences in how the COVID‐19 pandemic has negatively (top figure) and positively (bottom figure) affected their mental health

Girls (*M* = 2.819, *SD* = 1.792) reported a greater number of areas negatively affected by the COVID‐19 pandemic than boys (*M* = 1.728, *SD* = 1.687; *t*(501) = −10.787, *p < *.001). Significant gender differences emerged in terms of which factors associated with the COVID‐19 pandemic the youth considered to have a negative effect on their mental well‐being (Figure 3). Specifically, compared to adolescent boys, girls were significantly more likely to consider worrying about themselves (OR = 3.161, 95% CI 2.190–4.593, *p* < .001, Cohen's *d* = 0.635) and others close to them getting COVID‐19 (OR = 3.735, 95% CI 2.320–6.204, *p < *.001, Cohen's *d* = 0.726), increased time at home (OR = 3.203, 95% CI 2.181–4.755, *p* < .001, Cohen's *d* = 0.642), not being able to see friends in person (OR = 3.161, 95% CI 2.190–4.593, *p* < .001, Cohen's *d* = 0.635), worrying about people getting infected worldwide (OR = 3.735, 95% CI 2.320–6.204, *p* < .001, Cohen's *d* = 0.726), change in the school routine (OR = 3.203, 95% CI 2.181–4.755, *p* < .001, Cohen's *d* = 0.642), increased time with family (OR = 3.161, 95% CI 2.190–4.593, *p* = .001, Cohen's *d* = 0.635), increased stress due to change in daily routines (OR = 3.735, 95% CI 2.320–6.204, *p* < .001, Cohen's *d* = 0.726), and the news coverage of the pandemic (OR = 3.203, 95% CI 2.181–4.755, *p* < .001, Cohen's *d* = 0.642).

Overall, girls (*M* = 3.237, *SD* = 2.702) rated more factors associated with the COVID‐19 pandemic to have a positive effect on their mental health than boys (*M* = 2.622, *SD* = 2.702; *t*(473) = −2.557, *p = *.011). Gender differences also emerged in terms of what aspects of the COVID‐19 pandemic positively affected adolescent mental health (Figure 3). Specifically, girls were more likely than boys to rate increased time with family (OR = 1.928, 95% CI 1.320–2.837, *p* = .001, Cohen's *d* = 0.362), using social media (OR = 1.681, 95% CI 1.114–2.563, *p* = .014, Cohen's *d* = 0.286), watching TV (OR = 1.635, 95% CI 1.097–2.459, *p* = .017, Cohen's *d* = 0.271), and sleeping (OR = 1.567, 95% CI 1.097–2.246, *p* = .014, Cohen's *d* = 0.248), as well as increased flexibility in the daily routine (OR = 2.319, 95% CI 1.494–3.66, *p* < .001, Cohen's *d* = 0.464) as positively affecting their mental health. Boys, on the other hand, were significantly more likely than girls to consider decreased stress due to school and schoolwork (OR = 0.643 95% CI 0.430–0.961, *p* = .031, Cohen's *d* = −0.234) as positively affecting their mental health. No gender differences were noted in terms of increased time to relax, increased time for hobbies, increased time for exercising, less time with peers or news coverage of the COVID‐19 pandemic (*p > *.050).

## DISCUSSION

This study is among the first to investigate gender differences in the context of the broad‐scale effect of the COVID‐19 pandemic and associated social restrictions on adolescents and sheds light on which behavioral changes contribute to mental health problems due to COVID‐19. Overall, both adolescent boys and girls indicated that the COVID‐19 pandemic negatively impacted their lives, including their mental and physical health, peer relationships, academic performance, and overall day‐to‐day life. In addition, a third or more of adolescent girls and boys in our study identified change in their school routine, worrying about someone they know contracting COVID‐19, and not seeing friends in person during the pandemic as negatively affecting their mental health. Conversely, increased time for relaxing, sleeping, and hobbies were most commonly reported as having a positive impact on mental health. These findings highlight the consistent pursuit—and importance—of social activity for this age group. As such, during strict social restrictions, school administrators, healthcare providers, and parents should encourage and facilitate active social communication among peers.

In terms of gender differences, girls reported that the COVID‐19 pandemic and associated social restrictions had an overall greater negative impact on their lives compared to boys. Specifically, our findings demonstrate that girls were more likely than boys to perceive that the pandemic negatively affected their overall day‐to‐day lives, academic performance, and mental health. Consistent with the girls' perceived worsening in mental health outcomes, we found an increase of depressive symptoms, above and beyond expected levels based on previous years, among girls during the pandemic but not boys. The high ratings in the perceived negative impact of COVID‐19 on functioning among girls are undoubtedly intertwined with the increased depressive symptoms. However, given the cross‐sectional nature of this study, we cannot tease apart whether the depressive symptoms were skewing how adolescent girls viewed the effect of COVID‐19 on their lives or whether the broad‐scale negative effect of the pandemic was contributing to the depressive symptoms. The relationship could also be reciprocal, with both the broad‐scale overall disruption in their lives due to COVID‐19 increasing depressive symptoms and, in turn, the increased depressive symptoms adding to their bleak view of how their life has been affected by the pandemic. Nonetheless, the current findings provide insight into what areas of intervention may need to be prioritized for girls to mitigate the negative impact of the pandemic. However, more objective measures of the short‐term and long‐term impacts on physical health and academic performance are needed to decipher whether girls are indeed more negatively affected by the pandemic.

Interestingly, despite the increase in depressive symptoms among girls, we found no evidence for an increase in suicide attempts and anger during COVID‐19 among either girls or boys. These findings are consistent with previous studies leveraging national registry databases and clinical admission rates conducted in Japan (Isumi et al., 2020) and the United States (Mourouvaye et al., 2020). Although reassuring that the findings do not suggest an increase in suicide attempts, this is an important public health concern that needs to be monitored closely in the wake of the current pandemic. It is possible that a delayed effect may occur as adolescent girls who experienced increased depressive symptoms during COVID‐19 transition into young adulthood (Pierce et al., 2020).

Our findings also suggest that adolescent boys have been negatively affected by the pandemic, albeit to a lesser degree than girls. For instance, approximately a third or more of the boys expressed that the pandemic had negatively affected their academic performance, day‐to‐day life, peer relationships and physical and mental health. Yet, boys reported comparable levels of mental health problems during COVID‐19 to pre‐COVID‐19 ratings. Decreased sleep, online gaming with others, increased online gaming alone, and engaging with friends via social media during the pandemic all predicted higher depressive symptoms among boys. These findings highlight the need to determine the differential effects of stress and its contributors among adolescent girls and boys.

Online interactions have played an increasingly larger role in teens' social landscape in recent years (Odgers et al., 2020). This may be especially true during the pandemic. However, our findings suggest that the form of online interactions makes a difference in terms of whether it is associated with good or poor mental health for adolescent boys and girls. For instance, online gaming alone and active social media use (i.e., increased engagement with friends via social media) among boys and passive social media use among girls were associated with increased depressive symptoms. These findings are in line with research conducted elsewhere in the world (Ellis et al., 2020). Taken together, policymakers and health care professionals should recommend that parents monitor the quality of online interactions and encourage youth to actively participate in online social interactions in meaningful and productive ways.

There were also important gender differences in what factors associated with COVID‐19 were considered to positively affect adolescent reported mental health. Girls were more likely to consider increased time spent sleeping, time spent with the family, and relaxing during the pandemic to positively affect mental health compared to boys. Compared to girls, boys were more likely to consider less structure (e.g., more time for hobbies and to relax and decreased stress due to school) during the pandemic as having a positive effect on their mental health.

### Strengths and limitations

Among the strengths of the study is the extensive assessment of adolescent well‐being and the ability to compare the prevalence rates of mental health problems during the pandemic to recent nationwide averages. The use of self‐report measures to assess the perceived effect of COVID‐19 on adolescent outcomes can be viewed as both a strength or a weakness; however, we considered it a strength given that many adolescents with mental health problems do not seek help from healthcare providers (Kidger et al., 2012) and there is also evidence that adolescents are more likely to report mental health problems (e.g., suicide attempts) under conditions of anonymity (Safer, 1997). Moreover, these initial impressions of what areas adolescents perceive as negatively affected by the pandemic and what recent behavioral changes have occurred in response may inform potential intervention priorities, as well as inform what aspects of adolescent' needs to be tracked over time. Self‐report measures, on the other hand, are biased to the individual's mood. As such, more objective measures of well‐being and academic performance are needed to fully determine the effect of COVID‐19.

Additionally, only 22% of the original LIFECOURSE sample participated in the current study. There are several potential explanations for the low participation rate. First, approximately 20% of the participants from the original LIFECOURSE study had moved or did not have a valid home address listed within the school system (*n* = 448). Second, there was no central route (e.g., through the elementary schools) to contact all participants as they had graduated elementary school—which is mandatory in Iceland—and were enrolled in various secondary schools or had joined the workforce. Third, this was further complicated by secondary schools being limited to online teaching, making it harder to advertise the study within the schools, and there was general unrest within the Icelandic society due to frequent COVID‐19 infection flare‐ups and subsequent quarantine measures.

It is also important to note the limitations of cross‐sectional studies in that findings are based on associations and thus causal relationships cannot be claimed. Similarly, the possibility that other factors unrelated to the pandemic (e.g., the introduction of new social media outlets) may have contributed to the study findings cannot be excluded. Caution is also advised when interpreting the findings as boys were underrepresented in this study compared to prevalence rates in the general population of 16‐17‐year‐old adolescents. However, our findings indicate similar levels of self‐reported depressive symptoms among 16‐year‐old boys prior to and during COVID‐19 are consistent with that of our recent nationwide study, although elevated depressive symptoms were noted among 17‐year‐old boys (Thorisdottir et al., 2021). As such, further investigation into the well‐being of boys during COVID‐19 is needed. Moreover, our findings are based on youth who define themselves as female or male; thus, although the survey included the option of non‐binary definition of gender, given the small sample size (*n* = 2) of gender‐nonconforming youth, we were unable to conduct any meaningful analyses on this important subgroup. Further research is needed on gender‐nonconforming youth as research has indicated that they are already at heightened risk for a range of mental health conditions, which may be exacerbated during the pandemic (Hawke et al., 2021). Finally, the psychometric properties of the pandemic‐related survey items developed for this study have not been studied; however, they were based on existing international surveys on the effect of COVID‐19 on youth. Despite these limitations, we believe that the findings of this study add valuable information and advance our understanding of how adolescents perceive their well‐being in these unprecedented times and what intervention targets and strategies may prove effective.

## CONCLUSION

This study is among the first to examine gender differences in the broad‐scale adolescent response to COVID‐19 and potential contributors to such differences. The negative effect of the pandemic was more pronounced in girls, as they perceived the pandemic as having a more drastic and global impact on their lives compared to boys. Taken together, the findings suggest that a steady routine and remaining socially connected through telephone and online interactions may help youth—and especially girls—cope with the uncertainty and social restrictions associated with a pandemic. Moreover, healthcare providers, teachers, and other professionals should pay close attention to depressive symptoms, especially among girls during and after COVID‐19.

## CONFLICT OF INTERESTS

The authors declare that there are no conflict of interests.

## AUTHOR CONTRIBUTIONS

Thorhildur Halldorsdottir conceived the research question and design, conducted all analyses, and wrote the first draft of the manuscript. John P. Allegrante reviewed the first draft of the manuscript and was the first to provide feedback. Thorhildur Halldorsdottir, Ingibjorg Eva Thorisdottir, Bryndis Bjork Asgeirsdottir, Heiddis B. Valdimarsdottir, Alfgeir Logi Kristjansson, and Inga Dora Sigfusdottir determined the measurements used the surveys and coordinated its administration. Caine C. A. Meyers assisted with the literature review, reviewed drafts, and edited the manuscript. All authors reviewed drafts and edited the manuscript and approved the final draft.

## ETHICS STATEMENT

The present study adhered to the Helsinki Declaration. For each participant, one caregiver provided informed consent via electronic/bank ID and the adolescent provided assent prior to completing the survey in RedCap. The study was approved by the National Bioethics Committee of Iceland (11‐078) and registered with the Personal Protection Authority.

## Supporting information

Supplementary Material

## Data Availability

The LIFECOURSE study has an open‐data policy that is designed to foster collaboration with other research groups. For interested researchers, collaboration requests should submit proposed aims of the research and be addressed to the study's Principal Investigators, Inga Dora Sigfusdottir (ingadora@ru.is) or Thorhildur Halldorsdottir (thorhildurh@ru.is). The proposed study aims and potential overlap of requests with ongoing studies are discussed by the LIFECOURSE investigator team. Once approved, a LIFECOURSE team member will be assigned to supervise the collaboration and assist in conducting the project analysis.
