# Supplementary material for: Adolescent well‐being amid the COVID‐19 pandemic: Are girls struggling more than boys?
Source: JCPP Adv. 2021 Aug 3;1(2):e12027. doi: 10.1002/jcv2.12027 (PMC8420409; doi:10.1002/jcv2.12027)
Supplement: Supplementary file 1 — Supplementary Material [file JCV2-1-e12027-s001.docx]

**Supporting Information**

**Table S1**. COVID-19 survey items on behavioral change.

**Compared to prior to the pandemic, how much time do you spend in the following activity?**

Response scale: much less time; slightly less time; no change; slightly more time; much more time.

1. Sleeping
2. Physical activity
3. Watching movies, TV shows and videos
4. Meeting up with friends
5. Talking to friends on the phone
6. Use social media (e.g., Facebook, Snapchat, Instagram, TikTok) to talk with friends or send them a message, picture or video
7. Use social media (e.g., Facebook, Snapchat, Instagram, TikTok) to look at the profiles of friends
8. Meet up with relatives
9. Talk with relatives on the phone or through social media (e.g. Facebook, Snapchat, Instagram)
10. Use social media (e.g., Facebook, Snapchat, Instagram, TikTok) to look at the profiles of people you do not know
11. Use the internet for something else than social media or gaming (e.g., read news, watch videos)
12. Play computer games online alone
13. Play computer games online with others
14. Play computer games offline
15. Use the computer for something else than browsing online or playing computer games (e.g., study, write)
16. Watch the news

**Table S2.** Survey items on the perceived negative and positive effect on mental health due to the COVID-19 pandemic.

**What, if anything, do you feel has negatively affected your mental health during COVID-19?**

**Check all the items that apply to you.**

Worrying that I will get COVID-19

Worrying about someone I know getting COVID-19

Increased time at home

Not being able to meet friends in person

Worrying about how many people, in Iceland and abroad, have been seriously sick due to COVID-19

Changes in teaching and school routines

Increased time with the family

Stress due to changes in my daily routine

Media coverage of the COVID-19 pandemic

**What, if anything, do you feel has positively affected your mental health during COVID-19?**

**Check all the items that apply to you.**

Less stress due to school and homework

More time to relax

More time for hobbies (e.g., drawing, listening to music, writing, cooking)

More time using social media (e.g., Facebook, Snapchat, TikTok)

More time to watch TV

More time to exercise

Increased sleep

More time with the family

Less time with peers in school

Increased flexibility in my daily schedule

Media coverage of the COVID-19 pandemic

**Table S3.** Predictors of depressive symptoms during the COVID-19 pandemic across participants and stratified by gender.

|  | **All** | | | | **Girls** | | | | **Boys** | | | |
| --- | --- | --- | --- | --- | --- | --- | --- | --- | --- | --- | --- | --- |
| **Predictor** | **B** | **SE** | **P-value** | **Cohen´s d** | **B** | **SE** | **P-value** | **Cohen´s d** | **B** | **SE** | **P-value** | **Cohen´s d** |
| Sleep | -0.006 | 0.427 | 0.989 | -0.001 | 0.059 | 0.542 | 0.913 | 0.005 | -1.396 | 0.642 | 0.031 | -0.097 |
| Exercising | -0.585 | 0.393 | 0.137 | -0.066 | -0.581 | 0.509 | 0.255 | -0.051 | -0.225 | 0.569 | 0.693 | -0.018 |
| Watching TV | 0.684 | 0.526 | 0.194 | 0.058 | 0.042 | 0.671 | 0.951 | 0.003 | 1.280 | 0.780 | 0.103 | 0.073 |
| Meeting up with friends | -0.402 | 0.454 | 0.377 | -0.039 | -0.267 | 0.577 | 0.644 | -0.021 | 0.998 | 0.698 | 0.155 | 0.064 |
| Talking with friends on the phone | -0.365 | 0.556 | 0.512 | -0.029 | -0.908 | 0.685 | 0.186 | -0.059 | 0.455 | 0.847 | 0.592 | 0.024 |
| Engaging with friends via social media | 1.182 | 0.708 | 0.096 | 0.074 | 0.064 | 0.899 | 0.944 | 0.003 | 2.129 | 1.123 | 0.060 | 0.084 |
| Looking at friend's social media profile | 0.391 | 0.751 | 0.603 | 0.023 | 1.936 | 0.958 | 0.044 | 0.090 | -1.965 | 1.052 | 0.064 | -0.083 |
| Meeting up with family | 0.624 | 0.494 | 0.207 | 0.056 | 0.301 | 0.639 | 0.639 | 0.021 | 0.603 | 0.680 | 0.376 | 0.039 |
| Connecting with family via phone or social media | -1.795 | 0.586 | 0.002 | -0.137 | -2.143 | 0.778 | 0.006 | -0.123 | -1.054 | 0.824 | 0.203 | -0.057 |
| Looking at strangers' social media profiles | 1.767 | 0.616 | 0.004 | 0.128 | 0.903 | 0.756 | 0.233 | 0.053 | 0.837 | 0.999 | 0.404 | 0.037 |
| Non social media or gaming internet usage | 0.145 | 0.623 | 0.817 | 0.010 | -0.310 | 0.807 | 0.702 | -0.017 | 1.133 | 0.878 | 0.199 | 0.057 |
| Online gaming alone | 0.636 | 0.765 | 0.407 | 0.037 | 0.172 | 1.236 | 0.890 | 0.006 | 2.311 | 0.863 | 0.008 | 0.119 |
| Online gaming with others | -1.573 | 0.671 | 0.020 | -0.104 | -0.323 | 0.999 | 0.747 | -0.014 | -1.839 | 0.849 | 0.032 | -0.096 |
| Offline gaming | 0.245 | 0.687 | 0.721 | 0.016 | 1.037 | 0.978 | 0.290 | 0.047 | -0.456 | 0.886 | 0.608 | -0.023 |
| Computerized learning | -0.449 | 0.476 | 0.346 | -0.042 | -0.652 | 0.570 | 0.254 | -0.051 | -0.782 | 0.804 | 0.332 | -0.043 |
| New media coverage | 0.143 | 0.526 | 0.787 | 0.012 | -0.776 | 0.712 | 0.277 | -0.049 | -0.118 | 0.711 | 0.869 | -0.007 |

**Figures**

**Figure S1.** Distribution of the responses to the perceived broad-scale effect of COVID-19 by gender.

**Figure S2.** Distribution of the responses to self-reported behavioral change during the COVID-19 by gender.
